# Functional Validation of Endogenous Redox Partner *Cytochrome P450 Reductase* Reveals the Key P450s *CYP6P9a*/-*b* as Broad Substrate Metabolizers Conferring Cross-Resistance to Different Insecticide Classes in *Anopheles funestus*

**DOI:** 10.3390/ijms25158092

**Published:** 2024-07-25

**Authors:** Sulaiman S. Ibrahim, Mersimine F. M. Kouamo, Abdullahi Muhammad, Helen Irving, Jacob M. Riveron, Magellan Tchouakui, Charles S. Wondji

**Affiliations:** 1 Department of Biochemistry, Bayero University, Kano PMB 3011, Nigeria; 2 Center for Research in Infectious Diseases (CRID), Yaoundé P.O. Box 13591, Cameroon; mersimine.kouamo@crid-cam.net (M.F.M.K.); riveronjacob@gmail.com (J.M.R.); magellan.tchouakui@crid-cam.net (M.T.);; 3 Vector Biology Department, Liverpool School of Tropical Medicine (LSTM), Liverpool L3 5QA, UK; abdullahi.muhammad@lstmed.ac.uk (A.M.); helen.irving@lstmed.ac.uk (H.I.); 4 Center of Biotechnology Research, Bayero University, Kano PMB 3011, Nigeria

**Keywords:** *Anopheles funestus*, multiple, insecticides, resistance, *CYP6P9a*, *CYP6P9b*, endogenous, CPR

## Abstract

The versatility of cytochrome P450 reductase (*CPR*) in transferring electrons to P450s from other closely related species has been extensively exploited, e.g., by using *An. gambiae CPR* (*AgCPR*), as a homologous surrogate, to validate the role of *An. funestus* P450s in insecticide resistance. However, genomic variation between the *AgCPR* and *An. funestus CPR* (*AfCPR*) suggests that the full metabolism spectrum of *An. funestus* P450s might be missed when using *AgCPR.* To test this hypothesis, we expressed *AgCPR* and *AfCPR* side-by-side with *CYP6P9a* and *CYP6P9b* and functionally validated their role in the detoxification of insecticides from five different classes. Major variations were observed within the FAD- and NADP-binding domains of *AgCPR* and *AfCPR*, e.g., the coordinates of the second FAD stacking residue *AfCPR*-Y^456^ differ from that of *AgCPR*-His^456^. While no significant differences were observed in the *cytochrome c* reductase activities, when co-expressed with their endogenous *AfCPR*, the P450s significantly metabolized higher amounts of permethrin and deltamethrin, with CYP6P9b-AfCPR membrane metabolizing α-cypermethrin as well. Only the CYP6P9a-AfCPR membrane significantly metabolized DDT (producing dicofol), bendiocarb, clothianidin, and chlorfenapyr (bioactivation into tralopyril). This demonstrates the broad substrate specificity of *An. funestus CYP6P9a/-b*, capturing their role in conferring cross-resistance towards unrelated insecticide classes, which can complicate resistance management.

## 1. Introduction

Malaria, one of the deadliest diseases, infects 249 million people annually and killed 608,000 people in 2022 alone, 94% of these in sub-Saharan Africa [1]. Malaria control relies heavily on controlling the mosquito vectors which transmit the malaria parasite. Insecticide resistance in the major malaria vectors is threatening to derail the progress so far made in malaria control [2,3]. The resistance is mediated primarily through metabolic mechanisms [4], with *Anopheles* P450s from the CYP6 family taking the front seat in the detoxification of diverse insecticides from several public health classes [5,6,7,8,9,10]. For example, in the major malaria vector *An. gambiae*, several P450s have been shown to confer multiple resistance to more than one insecticide, e.g., *CYP6P3*, which alone confers resistance to pyrethroids, bendiocarb and pyriproxyfen [11,12,13]; *CYP6M2*, which alone confers resistance to pyrethroids, DDT, malathion, and pyriproxyfen [12,13,14,15]; and *CYP6Z2*, a P450 with broad substrate specificity, known to confer resistance to the primary products of pyrethroid hydrolysis [16,17], as well as pyriproxyfen [13].

In the major malaria vector, *An. funestus* studies have implicated P450s from the CYP6 family [18] in resistance towards several insecticide classes, e.g., the well-known pyrethroid metabolising specialists, *CYP6P9a* and *CYP6P9b* [19,20], which were shown recently to metabolize bendiocarb [21]; *CYP6Z1* and *CYP6AA1*, shown to confer pyrethroid and bendiocarb resistance [22,23]; and *CYP9K1*, recently shown to confer resistance to pyrethroids and DDT [24].

A key approach to validating the role of P450s in insecticide resistance has been through *in vitro* heterologous co-expression together with *CPR* in *Escherichia coli*, followed by HPLC-metabolism assays. This approach has been extensively exploited in *An. gambiae* and *An. funestus*, for example in the references cited above.

Cytochrome P450 reductase (*CPR*, EC 1.6.2.4) is an obligatory flavoprotein that plays a significant role in the metabolism of endogenous compounds and detoxification of exogenous xenobiotics such as drugs and pesticides/insecticides. Its pivotal public health importance is evident in its obligatory role for CYP450s, which are the most important superfamily of metabolic detoxification enzymes, mediating insecticide resistance [7,25,26,27], including in the *Anopheles* mosquitoes. The electron transfer from NADPH to CYP450s is facilitated by the *CPR*, which drives the rate-limiting step in many of the P450 enzyme-catalysed reactions [28]. The *CPR* shuttles electrons from NADPH through the FAD and FMN cofactors into the central heme iron of the P450s [29,30].

Previous studies have documented the contribution of *CPR* to insecticide resistance. For instance, in the malaria mosquito *An. gambiae*, an increase in the transcriptional levels of *CPR* has been observed in insecticide-resistant strains [8,12], in correlation with increased activity of known metabolic resistance-conferring P450s. Studies that silenced *CPR* in insects have documented reduced resistance, providing evidence for the direct role of *CPR* in insecticide resistance. For example, RNA interference (RNAi) silencing of *CPR* was shown to increase pyrethroid susceptibility in resistant populations of *An. gambiae* [31]. RNAi has been shown to significantly reduce the transcript levels of the *CPR* mRNA and increase adult susceptibility to malathion in the Oriental fruit fly, *Bactrocera dorsalis* [32]. Also, this approach has been shown to increase carbaryl susceptibility in the migratory locust, *Locusta migratoria* [33].

Several studies have utilised endogenous Anopheles *CPR* in *in vitro* assays, to characterise insecticide resistance-associated P450s. For example, the above-referenced studies for *An. gambiae* P450s, using *CPR* recombinantly co-expressed and characterised [11,12,13,14], and *sf9* expression of *An. minimus CPR*, together with *CYP6AA3* and *CYP6P7* to establish their roles in pyrethroid metabolism [34,35].

The ability of *CPR* to transfer electrons to P450s from closely related species has been exploited in several studies to establish the role of P450s in resistance. For example, *An. gambiae CPR*, hereafter *AgCPR* (sharing 96.5% percentage similarity to *An. funestus CPR*, hereafter *AfCPR*) has been utilised extensively as a homologous surrogate for *in vitro* functional validation of the role of *An. funestus* P450s in resistance towards pyrethroids [19,20,22,23,24,36,37], and recently bendiocarb [21]. However, it was observed recently that the two-spotted spider mite (*Tetranychus urticae*) *CYP392A16* (a P450 whose overexpression was shown to be linked with abamectin resistance) can only confer abamectin resistance *in vivo* (using transgenic *Drosophila melanogaster* flies) if co-expressed with its endogenous *CPR* [38]. This suggests that using surrogate *CPR* from other species may result in failure to detect potential metabolic activities toward specific insecticides/pesticides. Though a recent study has described the co-expression of *AfCPR* together with *CYP6P9a* and *CYP6P9b* in a Baculovirus system [39], it investigated the ability of these P450s to metabolise only deltamethrin. The study did not investigate the metabolism of non-pyrethroid insecticides or the risk of cross-resistance between unrelated insecticide classes that could potentially be conferred by these duplicated P450s. In contrast with *An. gambiae* P450s, the *An. funestus* P450s have not been shown to confer cross-resistance to more than three insecticide classes, probably due to the non-optimal metabolism assay conditions, e.g., utilization of *AgCPR* as a homologous electrons supply redox partner, even though the CPRs from these two species differs by 24 amino acids. We hypothesise here that *CYP6P9a/-b*, which are currently massively selected in field populations across Africa, could pose a greater risk to vector control by exhibiting a broad metabolic activity towards multiple insecticides, and that using the endogenous *AfCPR* could better capture and reveal this phenomenon.

Therefore, in this study, the key pyrethroid-metabolizing P450s, *An. funestus CYP6P9a* and *CYP6P9b*, were recombinantly co-expressed with endogenous *AfCPR*, alongside the homologous *AgCPR* in *E. coli* cells. The purified membranes were utilised for HPLC-based metabolism assays, showing that in the presence of endogenous *AfCPR*, these P450s can metabolise the pyrethroids deltamethrin and permethrin with higher depletion. In addition, membranes expressing *CYP6P9b* and *AfCPR* (hereafter CYP6P9b-AfCPR) metabolise α-cypermethrin. Of importance is the finding that the presence of *AfCPR* confers upon *CYP6P9a* the ability to significantly metabolise non-pyrethroid insecticides, including DDT (producing a hydroxylation product, dicofol/kelthane), clothianidin, and chlorfenapyr (which is bioactivated into insecticidally toxic primary metabolite tralopyril).

## 2. Results

### 2.1. Patterns of Genetic Variability of AfCPR

Analysis of the polymorphism patterns of full-length cDNA sequences of *AfCPR* (2040 bp) showed high homogeneity in sequences from Malawi and FANG (Angola), with their haplotypes clustering in the maximum likelihood phylogenetic tree (Figure S1a). In contrast, unusually high polymorphisms were observed in Uganda *CPR*, with its haplotype clustering separately, as an offshoot.

Except for the Uganda sequences, *AfCPR* exhibited low polymorphism, with only two haplotypes, one each in Malawi and FANG (Figure S1b and Table 1). Overall, from the 15 sequences, there were only four haplotypes, with only two haplotypes for Uganda sequences despite its unusually high polymorphism compared to the other sequences (Figure S1c). Four amino acid substitutions were observed in Uganda.

Haplotype diversity is high (H_d_ = 0.77), from 14 haplotypes out of 15 sequences. The very low H_d_ in the sequences from Malawi and FANG (Hd = 0.00, π = 0.00 for both) suggests a selective pressure and is consistent with the high sequence conservation known in *CPR*. The neutrality test of all sequences revealed Tajima’s D and Li and Fu’s D* as positive and statistically significant. The statistic was also positive for Uganda sequences, with Li and Fu’s D* significant, suggesting rare polymorphism and lack of background selection.

### 2.2. Comparative Sequence Characterisation of Anopheles CPR

Analysis of the 2040 bp open reading frame of *AfCPR* revealed that it is 96.5%, 91.86%, and 89.37% identical to *An. gambiae* (GenBank: AY18963375), *An. minimus* (EF0957735), and *An. stephensi* (BK008720) *CPRs*, respectively.

**Figure 1:**
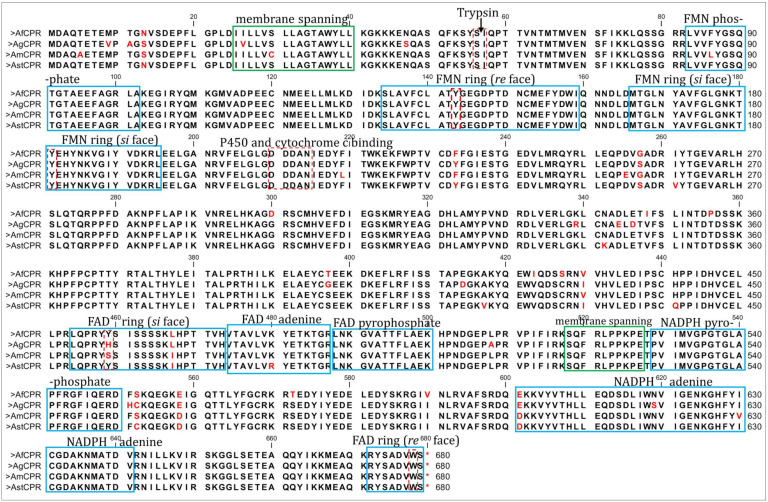
**Comparison of Anopheles *CPR* amino acid sequences.** Af, Ag, Am, and Ast refer to *An. funestus*, *An. gambiae*, *An. minimus*, and *An. stephensi*, respectively. Predicted membrane-spanning domains are in solid green boxes; the cofactor-binding regions are in solid blue boxes; and critically important residues are in red, dashed boxes. Variable amino acids are in red.

Comparative mapping of the various Anopheles *CPR* sequences to the rat *CPR*, and Pfam server predictions revealed high conservation in the structural and functional domains. Initial prediction of the transmembrane domain using DeepTMHMM predicted Anopheles *CPR* residues 25–40 as the membrane-spanning amino acids (Figure S2). These sequences are identical between *AfCPR* and *AgCPR*. Within the hydrophobic terminal membrane binding domain, the uniquely sensitive trypsin-cleavable Lys^56^/Ile^57^ bond purported to demarcate the membrane-binding segment from the catalytic portion of the *CPR* [40] is conserved as Ser^56^/Ile^57^ in all Anopheles *CPR* sequences (Figure 1).

The FMN-binding domain is also highly conserved between the Anopheles species, with the FMN-binding polypeptide fragments T^141^Y^142^G^143^E^144^G^145^D^146^P^147^ and N^178^K^179^T^180^Y^181^E^182^H^183^F^184^N^185^ [41] being identical. The phenolic rings of the two critical tyrosine residues, Y^140^ (Y^143^ in Anopheles sequence) at the *re*-side and Y^178^ (Y^181^ in Anopheles) at the *si*-side, which sandwich the juxtaposed flavin isoalloxazine rings and are known to be critical to FMN-binding and catalysis [41,42], are conserved in all Anopheles, as in the rat *CPR*. Additionally, the loop between strand 5 and helix F (containing the docking site acidic residues 210–2014, D^210^D^211^D^212^A^213^N^214^) described as being involved in binding to P450s and cytochrome *c* [43,44], is conserved in all the Anopheles species. In short, within the 70–225 residues encompassing the FMN-binding region, no major changes in co-factor binding regions were observed, with phosphate moiety, FMN ring (*re* face), and *si* face identical in the Anopheles sequences.

However, major variations were observed in the connecting domain (encompassing residues 240–270 and 325–450), which is situated between the FMN-binding domain and the FAD- and NADPH-binding domains and which is responsible for orienting these two domains, ensuring proper alignment of the two flavin moieties necessary for electron transfer [41]. For example, within the first connecting domain, G^257^ in *An. funestus* and *An. minimus* is replaced with Ser in *An. gambiae* and *An. stephensi*. In addition, within the highly conserved second connecting domain, 7 amino acid variations (residues 344, 346, 348, 356, 396, 414, and 427) were observed, with *AfCPR* remarkably different from *AgCPR*. For example, G^396^ in *An. gambiae* is T^396^ in *An. funestus*, while Ser occupies this position in the rest of the sequences. Serine residue occupies position 427 (S^427^) in *An. funestus*, while the rest of the sequences have Cysteine in this position.

Subtle variations between *AfCPR* and *AgCPR* were also observed in the FAD binding domain (encompassing residues 450–520). The *re* face of the FAD ring (R^672^Y^673^S^674^A^674^D^675^V^676^W^677^S^678^) is stacked with the indole ring of W^678^, a residue conserved in all species, and the Y^459^ aromatic ring (Y^459^Y^460^S^461^I^462^S^463^S^464^ fragment), a residue which stacks the FAD ring in the *si* face, and which is identical in rat *CPR*, *AstCPR*, and *AfCPR* but replaced with H^459^ in *AgCPR* and S^459^ in *AmCPR* [41]. The pyrophosphate is stabilized by the side chain of T^494^ and R^454^ which are conserved in all species. The phenolic ring of Y^481^ (immediately downstream of the V^474^T^475^A^476^V^477^L^478^V^479^ fragment encompassing the FAD), which stacks on one side of the adenine ring, is conserved in all species.

For the NADP binding domain (residues 522–679), the loop S^518^Q^519^F^520^R^521^L^522^P^523^P^524^K^525^P^526^E^527^ purported to be a membrane binding domain is conserved in all the Anopheles. However, residue variations were observed within this domain. For example, S^552^ in *AfCPR* and *AmCPR*, with Cys in the same position in *AgCPR* and *AstCPR*; position 572, where all sequences have Ser while *AfCPR* has T^572^; position 590, with *AfCPR* having V^590^ while all other sequences have I^590^; and N^619^ in all sequences except for *AgCPR* with Ser in the same position. The conserved residues associated with 2’-phosphate binding in human *CPR* (S^597^, R^598^, K^603^ and K^635^ [45,46,47]) are identical in all the Anopheles species.

### 2.3. Comparative Prediction of AfCPR and AgCPR 3D Folding and Interaction with AfCYP6P9a

To investigate differences in the 3D folding conformation of *AfCPR* and *AgCPR*, models of the proteins were created. A total of 12 models were generated for each sequence, and Figure S3 summarizes the internal structure assessments with DOPE in tabular form (panels a and d), as well as the comparative DOPE energy profile between the template 1AMO and the models with the highest quality (panels b and e). Ramachandran energetic validation revealed that 489 residues (90.6%), 46 residues (8.5%), and 2 residues (0.4%) were in the most favoured regions, additionally allowed regions and generously allowed regions for AfCPR model number 2 (panel c), with only 3 residues (0.6%) in disallowed regions. For AgCPR model number 7, 497 residues (92%), 39 residues (7.2%) and 1 residue (0.2%) were in the most favoured regions, additionally allowed regions, and generously allowed regions, with only 3 residues (0.6%) in the disallowed regions (panel f). External assessments using Errat revealed overall quality scores of 74.21% and 74.38% for the AfCPR model 2 and AgCPR model 7, respectively (Figure S4). The AfCYP6P9a model has an overall Errat quality score of 49.48% (Figure S5d) and Ramachandran assessment predicted that 366 residues (85.3%), 46 residues (10.7%), and 8 residues (1.9%) were in the most favoured regions, additionally allowed regions, and generously allowed regions, with 9 residues (2.1%) in disallowed regions (Figure S5c).

**Figure 2:**
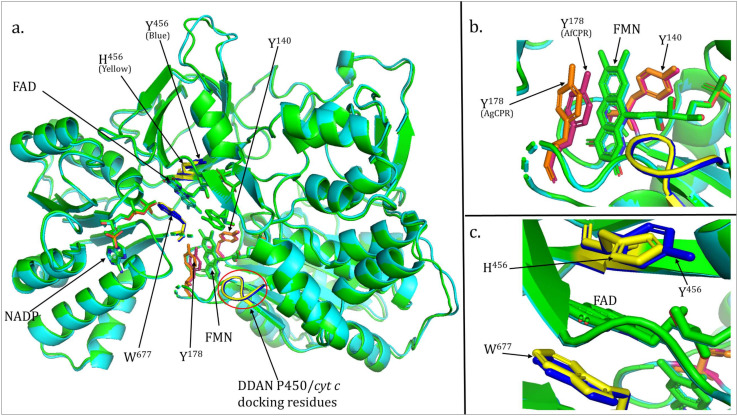
**Three-dimensional folding of AfCPR and AgCPR models.** (**a**). Overlay of AfCPR and AgCPR models. Conserved critical residues and cofactors are in stick format and labelled. Residues are in warm pink and blue in AfCPR, and in orange and yellow in AgCPR. The DDAN-P450/*cytochrome c* binding sequence residues are highlighted in blue in AfCPR and yellow in AgCPR models. (**b**). Close shot of the FMN stacking residues showing a contrasting coordinate in Y^178^ between AfCPR and AgCPR models. (**c**). Close shot of the FAD stacking residues showing the contrasting coordinates of Y^456^ in AfCPR and H^456^ in AgCPR models.

Overall, the two CPR models exhibited similar folding patterns, with the critical aromatic residues known to stack and stabilize the FMN and FAD rings in close proximity to their cofactors (Figure S6). However, the overlay of the two models revealed subtle differences that could potentially lead to differences in catalysis between the two proteins. For example, while the FMN-sandwiching residue Y^140^ (Y^143^ in Anopheles) exhibits similar coordinates, the coordinates of the aromatic ring of the second FMN-stacking residue Y^178^ (Y^181^ in Anopheles sequences) differ between the AfCPR and AgCPR models (Figure 2a,b).

Also, while the coordinate of the indole ring of the critical W^677^ residue (W^678^ in Anopheles sequences), known to stack the FAD ring, is similar, the coordinate of the second FAD stacking residue Y^456^ (Y^459^ in AfCPR) differs from the coordinate of His^456^ in the AgCPR model (Figure 2a,c).

**Figure 3:**
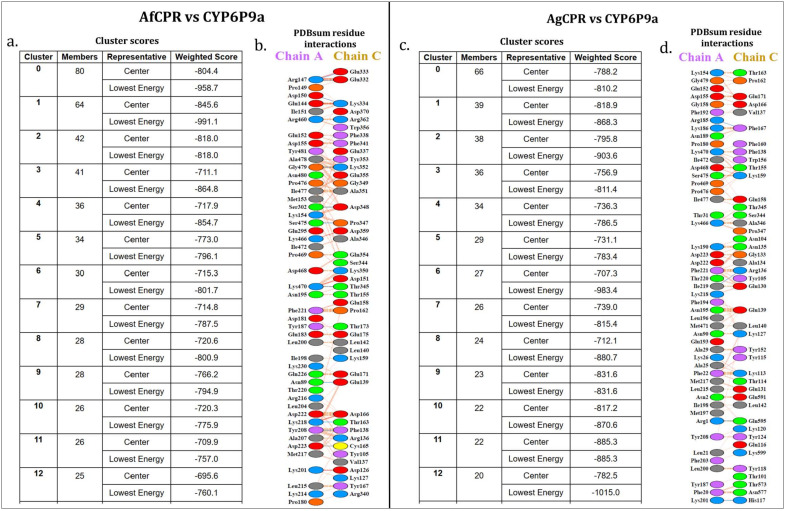
**ClusPro docking scores and PDBsum prediction of residues involved in CPR−P450 interactions.** Note: The crystal templates of 1TQN for CYP6P9a and 1AMO for CPR lack residues 1–22 and 1–66, respectively. Amino acid counts are thus +22 for Chain A (CYP6P9a) and +66 for Chain C (AfCPR/AgCPR). Blue lines are hydrogen bonds, and red lines are salt bridges.

To investigate potential variations in interactions between insecticide resistance-conferring P450s and the redox partners AfCPR and AgCPR, comparative docking simulations were conducted with the CPR models and a model of a well-known metabolic resistance P450, *CYP6P9a*. Docking established that *AfCPR* and *CYP6P9a* interaction produces 80 cluster members in its most productive pose, with a lowest weighted energy score of −958.7, compared with the interaction of *AgCPR* with *CYP6P9a*, with 66 cluster members and a weighted energy score of −810.2 (Figure 3a,c). Protein-protein interaction analysis revealed a striking difference between the intermolecular interactions of the two CPR models with the CYP6P9a model. A total of 47 residues and 44 residues were predicted to be involved in interactions in CYP6P9a and AfCPR, respectively (the productive cluster with the lowest weighted energy score), with 7 salt bridges and 11 hydrogen bonds predicted, within 612 non-bonded contacts (Figure 3b). A total of 49 residues and 43 residues were predicted to be involved in interactions in CYP6P9a and AgCPR, respectively, with 4 hydrogen bonds predicted, within 564 non-bonded contacts (Figure 3d). No salt bridges were predicted in this interaction.

Residues involved in the 11 hydrogen bonds between CYP6P9a and the AfCPR models include R^169^, located downstream of the α-helix of *AfCPR*, and hydrogen bonded to E^398^ and E^399^, which are two residues located within the J helix in the connecting domain of *AfCPR* (Figure 3b). N^111^, which is a second residue within the substrate recognition site 1 of *CYP6P9a* [refer to [19] for the topology of the structurally conserved regions of *CYP6P9a*], is hydrogen bonded to E^205^ (β sheet 5), an amino acid located 5 residues downstream of the DDAN motif of *CPR* (composed of acidic residues known to be the docking site of P450s and *cytochrome c*). Also, another residue involved in hydrogen bonding is D^245^, located within SRS-3 of *CYP6P9a* and hydrogen bonded to C^231^, another residue located downstream of the DDDAN motif.

The same residue above, CYP6P9a-R^169^, was predicted to be involved in salt bridges to AfCPR- E^398^ and -E^399^. Other interactions from the seven predicted salt bridges include a bridge between CYP6P9a-K^492^ to AfCPR-D^217^, located two residues downstream of the DDDAN motif; and CYP6P9a-K^223^, located five residues downstream of SRS-2, bridged to D^192^, a residue located within the FMN ring (*si* face).

For the AgCPR model, five hydrogen bonds were predicted, including between K^223^ (located five residues downstream CYP6P9a SRS-2) and H^183^ (located within the FMN ring, *si* face) (Figure 3d); between I^219^ located within *CYP6P9a* SRS-3 and R^202^ (located between the FMN ring, *si* face, and the DDDAN motif); and R^207^, located three residues upstream of the *CYP6P9a* SRS-2, hydrogen bonded to AgCPR-F^233^, and located within the first connecting domain separating the FMN- from the FAD-binding domains.

### 2.4. Comparative Assessment of Cytochrome c Reductase Activity

The recombinant AfCYP6P9a and -b expressed optimally 36–40 h after induction, with P450 spectral concentrations of 16.82 nmol/mL, 17.81 nmol/mL, 15.44 nmol/mL, and 13.17 nmol/mL for CYP6P9a-AfCPR, CYP6P9a-AgCPR, CYP6P9b-AfCPR and CYP6P9b-AgCPR membranes, respectively. The total protein concentrations were 27.8 mg/mL, 26.1 mg/mL, 20.8 mg/mL, and 26.6 mg/mL, respectively.

**Figure 4:**
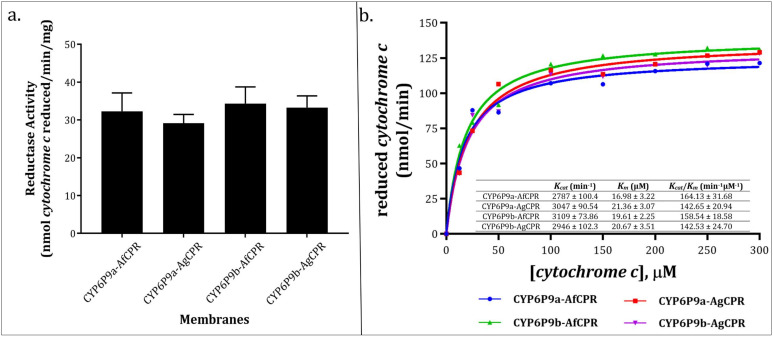
**Comparative reductase activity of recombinant CPR proteins.** (**a**). Initial assessment of *cytochrome c* reductase activity. (**b**). Michaelis–Menten plot of *cytochrome c* reduction by recombinant AfCPR and AgCPR co-expressed side-by-side with CYP6P9a and CYP6P9b.

Initial assessment of *cytochrome c* reductase activity revealed 32.27 ± 4.85, 29.2 ± 2.31, 34.32 ± 4.49, and 33.27 ± 3.09 nmol *cytochrome c* reduced/min/mg protein for the membranes expressing CYP6P9a-AfCPR, CYP6P9a-AgCPR, CYP6P9b-AfCPR, and CYP6P9b-AgCPR, respectively (Figure 4a), suggesting comparable CPR activities.

Reduction of *cytochrome c* follows Michaelis–Menten fashion, with comparable maximal velocities of 125.4 ± 4.59, 137.1 ± 4.07, 139.9 ± 3.23, and 132.6 ± 4.60 nmol *cytochrome c* reduced/min. The catalytic constant (*K_cat_*) was comparable, within the ranges of 2787–3109 min^−1^, with the CYP6P9a-AfCPR membrane producing the lowest rate (Figure 4b). However, the CYP6P9a-AfCPR membrane exhibited the comparable but highest affinity for *cytochrome c*, with *K_m_* of 16.98 µM, translating into the highest catalytic efficiency of 164.13 min^−1^ µM^−1^. Overall, no significant differences in terms of *cytochrome c* metabolism were observed between the two recombinant CPRs.

### 2.5. Comparative Assessment of the Role of AfCPR on Insecticide Metabolism by Recombinant CYP6P9a/-b

**Figure 5:**
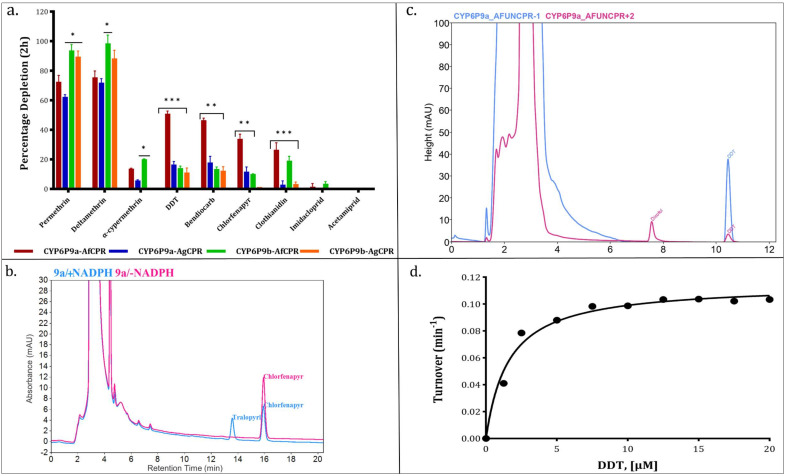
**Comparative profiling of impact of AfCPR on metabolism of insecticides**. (**a**). Substrate−depletion by the recombinantly co-expressed CYP6P9a and -b with either AfCPR or AgCPR. *, ** and *** = significantly different at *p* < 0.05, *p* < 0.01 and *p* < 0.001, respectively. (**b**). HPLC chromatogram showing chlorfenapyr metabolism by CYP6P9a-AfCPR membrane, with tralopyril eluting before the 14th minute. (**c**). HPLC chromatogram showing DDT metabolism by CYP6P9a-AfCPR membrane, with dicofol eluting before the 8th minute. (**d**). Michaelis–Menten plot showing turnover of DDT metabolism by CYP6P9a-AfCPR membrane.

Substrate depletion assays conducted with permethrin and deltamethrin revealed high metabolism in CYP6P9a and -b membranes co-expressed with both CPRs, with the recombinant CYP6P9b depleting higher amount of both pyrethroids. For example, CYP6P9b-AfCPR and -AgCPR depleted 90% and 93% of permethrin (*p* < 0.05) compared with 73% and 62% in CYP6P9a-AfCPR and -AgCPR, respectively (Figure 5a). A similar pattern was seen in the case of deltamethrin, with depletion of 99% by the CYP6P9b-AfCPR, which is significantly higher than the 72% obtained with CYP6P9a-AgCPR (*p* < 0.05).

Low depletion was observed towards α-cypermethrin, less than 5% for both membranes of CYP6P9a and CYP6P9b when co-expressed with AgCPR. This depletion significantly increased to 14% and 21% in the CYP6P9a-AfCPR and CYP6P9b-AfCPR membranes (*p* < 0.05).

While no appreciable metabolism of DDT was observed (depletion of less than 15%) from CYP6P9b membranes co-expressed with either AfCPR or AgCPR, depletion of 17% was obtained from CYP6P9a-AgCPR, and a significant depletion of 51% (*p* < 0.001) by the recombinant CYP6P9a membrane co-expressed with the AfCPR. The CYP6P9a DDT metabolism proceeds with the oxidation of trichloromethyl carbon to produce dicofol (Figure 5c). Metabolism of DDT follows Michaelis–Menten fashion (Figure 5d), with a maximal velocity of 5.12pmol/min, *K_cat_* of 0.112 min^−1^ and *K_m_* of 1.59 µM, translating into catalytic efficiency of 0.07 min^−1^ µM^−1^.

A similar pattern was observed with respect to bendiocarb, with CYP6P9a-AfCPR and CYP6P9a-AgCPR significantly depleting 47% (*p* < 0.01) and 18% of this carbamate insecticide compared to less than 15% depletion obtained from CYP6P9b membranes. *CYP6P9a* metabolism of bendiocarb is like previous observations with other *An. funestus* P450s, e.g., recombinant CYP6Z1 and CYP9J11, which generate polar metabolites eluting at the beginning of the chromatogram [22,36].

On the other hand, only CYP6P9a membrane co-expressed with endogenous AfCPR membrane metabolised chlorfenapyr, significantly depleting 34% of this pyrrole insecticide (*p* < 0.01), compared to ~11% depletion obtained from CYP6P9a-AgCPR and CYP6P9b-AfCPR. CYP6P9a-AfCPR metabolism of chlorfenapyr generated a bioactive primary metabolite, tralopyril (Figure 5b).

For neonicotinoids, no metabolism was observed with acetamipirid for all membranes, and less than 5% of imidacloprid was depleted by all membranes. However, for clothianidin, significant depletion was obtained from both CYP6P9a (27% depletion, *p* < 0.01) and CYP6P9b (19.6%, depletion, *p* < 0.01) membranes co-expressing AfCPR, compared to the membranes expressing the same P450s but with AgCPR, which depleted less than 5% of this insecticide.

## 3. Discussion

Understanding the mechanisms of resistance and the patterns of cross-resistance between unrelated insecticide chemistries is critical to designing suitable resistance management plans, with the potential to reduce malaria burden. In this respect, deciphering the interactions of *CPR* and major metabolic resistance P450s, such as *CYP6P9a*/-*b* in *An. funestus*, is essential to better implement insecticide-based interventions.

The critical role of *CPR* is evident in its public health clinical importance. For example, in humans, *CPR* is involved in steroidogenesis, bone formation, and drug metabolism [48], and mutations in the human *CPR* result in reduced activities of steroid metabolising enzymes, *CYP17A1* (17, α-hydroxylase)/17,20 lyase), and *CYP19A1* (aromatase), causing a rare form of congenital adrenal hyperplasia characterised by adrenal insufficiency, genital anomalies, and bony malformations resembling Antley–Bixler syndrome [49,50].

Within the context of vector-borne diseases burden, *CPR* from the major malaria vectors have been implicated in contributing to insecticide resistance in several genome-wide transcriptional and functional validation studies [8,12,31].

### 3.1. Evidence of High Homology in AfCPR Sequences

Very low polymorphism was observed in the *CPR* sequences from FANG and Malawi. This is not surprising as *CPR*, occurring as a single copy, is a conserved protein whose functional domains are known to be highly conserved even across species, as even single mutations can lead to debilitating diseases in eukaryotes [51]. Despite the unusually high polymorphism observed in the Uganda sequences, only four amino acids substitution was observed, suggesting a high conservation in *An. funestus* despite the high cDNA polymorphism. However, more *AfCPR* sequences from across Africa need to be analysed to fully capture this pattern.

### 3.2. Homologous CPR May Not Reconstruct Full Detoxification Potentials of Certain P450s

The redox partner promiscuity of *CPR* has been exploited in several studies to investigate the role of P450s in metabolic resistance in the major malaria vectors. A typical example is *AgCPR*, which has been utilised extensively as a surrogate since it was first reported in 2006 [16] for *in vitro* functional validation of the role of *An. funestus* P450s in insecticide resistance [19,20,21,22,23,24,36,37]. However, the observation that *CYP392A16* can only confer abamectin resistance *in vivo* using transgenic *D. melanogaster* flies if it is expressed with its endogenous *Tetranychus urticae* (two-spotted spider mite) *CPR* [38], and the fact that studies have shown that even a single amino acid change (e.g., P280L in humans) can affect *CPR* stability, reducing activity and leading to lower testosterone levels [52], suggests that utilization of surrogate *CPR* from other species may result in failure to detect potential metabolic activities toward many substrates which can potentially be metabolised by P450s if co-expressed with their endogenous *CPR*. This means that there is a possibility that even a single amino acid variation, e.g., Y^456^ (*AfCPR*)/H^456^ (*AgCPR*), a critical residue which stacks and stabilises the FAD ring, could potentially modify activities. Indeed, in humans, the Y^456^/H^456^ mutation [53] has been shown to disrupt the binding of FAD and impair *CYP4A4* activity in patients afflicted with Antley–Bixler syndrome [54]. Also, a single amino acid change in rat liver *CPR* has been shown to modify the molecular weight of the recombinantly expressed CPR protein. For example, in SDS-polyacrylamide gels, wild-type rat *CPR* migrated as a single band of approximately 80,000 daltons, but replacement of the key residues Y^140^ to D^140^ (established as a requirement for FMN binding and catalysis) resulted in detectable alterations in the electrophoretic mobilities, with the protein exhibiting an anomalous molecular weight of 84,000 to 85,000 daltons [42]. This strongly suggests that even a single amino acid change can possibly alter the folding pattern, conformation, and/or activity of Anopheles *CPR*.

### 3.3. Recombinant AfCPR and AgCPR Exhibits Comparable Cytochrome c Reductase Activities

Comparative assessment of the metabolism of *cytochrome c* by the recombinant AfCPR and AgCPR revealed comparable activities between the four membranes (within the range of 29 and 34 nmol/min/mg). These values are within ranges established for other organisms. For example, CPR activities of 43.6–162.5 nmol/min/mg protein have been described for co-expressed CYP450/CPR membranes from *Culex quinquefasciatus* [55]. Another study which co-expressed *An. minimus CYP6AA3* with its endogenous *AmCPR* has described membranous activity of 41.16 nmol/min/mg protein [56], which was comparable to a follow-up study in the same species with *CPR* expressed singly as a membrane protein [57]. While 61 nmol/min/mg activity has been described for *AgCPR* previously [11], specific activity of up to 23.9 µmol/mg/min has been described for a purified *AgCPR* [47]. However, this Δ2-63 truncated protein (the membrane anchor sequence was deleted by removal of amino acids 2–63) was expressed singly and in soluble form.

Steady-state kinetic analysis of *cytochrome c* reduction revealed comparable catalytic constants for all membranes, in ranges of 2787 min^−1^ for CYP6P9a-AfCPR membrane to 3109 min^−1^ for CYP6P9b-AfCPR membrane. These values are very similar to 56.77 s^−1^/3.40 min^−1^ described for *AmCPR* [57], but are on average half the turnovers of 105 s^−1^ described for *AgCPR* and human *CPR* (88 s^−1^) [47].

For both recombinant AfCPR and AgCPR, *cytochrome c K_m_* values (17–21 µM) were very similar to the previously established values for Anopheles *CPR*s. For example, in the above studies, Lian and colleagues [47] reported *K_m_* values of 19 µM and 23 µM, respectively, for recombinant AgCPR and human CPR. Two other studies have independently confirmed *K_m_* values of 19.07 µM [57] and 27.39 µM [58] for *AmCPR*, further confirming similar binding affinities for cytochrome *c.* However, deviations from these include low *K_m_* values, including 2.58 µM described for *AmCPR* [56], and 1–4 µM described for housefly [59] and rat [60] *CPRs*. Overall, the recombinant AfCPR exhibited slightly higher catalytic efficiencies compared with those obtained with AgCPR, suggesting that both CPR catalyse *cytochrome c* reduction with similar profiles.

### 3.4. Endogenous CPR Confers Non-Pyrethroid Metabolising Abilities in CYP6P9a and -b

Pyrethroid metabolism assays reveal high depletion, especially with CYP6P9b, which metabolised more than 90% of deltamethrin and permethrin regardless of the reductase it was co-expressed with, consistent with our previous observations [19,20,37].

Assays with α-cypermethrin revealed no activity towards this type II pyrethroid, except for the marginal depletion of 21% obtained with CYP6P9b-AfCPR membranes. We considered the 20% substrate depletion cut-off value as significant since it is normally applied in drug screening to rule out metabolic activity from uncertain metabolism, potential binding, and baseline variability [61].

The finding of recombinant CYP6P9b metabolising α-cypermethrin is of grievous importance, as this insecticide is currently the bedrock of the most insecticidal next-generation, combination, long-lasting insecticidal bed nets, such as Interceptor G2 (composed of α-cypermethrin and chlorfenapyr) and Royal Guard (α-cypermethrin plus pyriproxyfen). Indeed, *An. gambiae CYP6P3* has been recently shown to deplete >80% of α-cypermethrin [62], and *CYP6P5* from the New World malaria vector *An. albimanus* has been shown to deplete 57.4% of this insecticide, metabolising it with high turnover and high efficiency [63]. The fact that CYP6P9b-AfCPR membrane can metabolise α-cypermethrin, in contrast with the lack of significant metabolism when co-expressed with AgCPR, suggests that the amino acid variations between the two CPRs could be playing a role in the docking and interaction of this P450 with the endogenous *CPR*. Indeed, studies have established the role of individual *CPR* amino acids in the metabolism of pyrethroids. For example, the L^86^F (FMN-binding domain) and L^219^F (4 residues downstream the DDAN motif) mutants of *AmCPR* were shown to exhibit increased FMN retention in the *AmCPR* and significantly increased deltamethrin degradation [57]. Similar changes in metabolic profiles were observed upon the introduction of additional mutations into the *AmCPR*. These findings strongly suggest that the 24-amino-acid difference between *AfCPR* and *AgCPR* could potentially lead to marked differences in metabolic profiles.

Our previous studies have shown that the recombinant CYP6P9a and -b co-expressed with AgCPR cannot metabolise non-pyrethroid insecticides, including DDT and bendiocarb [22,37]. However, the significant depletion obtained upon co-expression of *CYP6P9a* with its endogenous *AfCPR* indicates this P450 can biotransform these indoor residual spraying insecticides. Indeed, other studies have shown that P450s from the CYP6 family can detoxify DDT. For example, *An. gambiae CYP6M2* has been shown to metabolise DDT, though producing DDE, in addition to dicofol [14], in contrast to our observation with *CYP6P9a*, which generated dicofol only. Also, a recent study has demonstrated moderate DDT resistance in transgenic *Drosophila* flies expressing *An. albimanus CYP6P5* [63].

*CYP6P9a* can metabolise bendiocarb as well, but only in the presence of its endogenous *AfCPR*. In our previous studies, using *AgCPR* as a surrogate, we have shown that *An. funestus CYP6Z1* [22], *CYP6AA1* [23] and *CYP9J11* [36] can metabolise bendiocarb. Also, we have shown recently that *CYP6P9a* can metabolise bendiocarb when co-expressed with *AgCPR*, but with a depletion of only 18% [21]. *CYP6P3*, an ortholog from *An. gambiae*, has been demonstrated to confer a moderate bendiocarb resistance in transgenic *Drosophila* flies [12].

Several studies have vouched for the insecticidal efficacy of chlorfenapyr, especially against natural populations of *An. funestus*. These include, for example, a semi-field assessment using cone bioassays and experimental hut trials, which have shown the high efficacies of Interceptor G2 in killing both *An. gambiae* and *An. funestus* in several African countries [64,65,66]. However, a recent multi-country study has established a reduced susceptibility of chlorfenapyr in the field populations of *An. gambiae* from DRC, Ghana, and Cameroon, while full susceptibility was reported in *An. funestus* [67]. Other studies have also established chlorfenapyr resistance, for example in *An. gambiae* populations from several sites in Côte d’Ivoire [68]. Given that chlorfenapyr is primarily bioactivated by P450s into the insecticidally active ingredient tralopyril [69], which induces death in insects, it should be expected that *An. gambiae* populations should be highly susceptible to this insecticide through P450 bioactivation. However, the recent contrasting observation that key *An. gambiae* P450s, *CYP6P3* and *CYP9K1*, ubiquitously overexpressed across African populations of *An. gambiae* (shown in several studies above to be resistant to chlorfenapyr) bioactivate this pro-insecticide into its tralopyril toxic intermediate [62] calls for more in-depth characterisation of the role of *An. gambiae* P450s and/or other metabolic detoxification enzyme classes in chlorfenapyr biotransformation. Nevertheless, our finding that *An. funestus CYP6P9a* bioactivates chlorfenapyr into insecticidally active tralopyril is consistent with the widespread susceptibility of field populations of *An. funestus* toward this pyrrole insecticide, e.g., [64,70,71].

Clothianidin has found utility as a front-line indoor residual spraying ingredient, for example in Fludora Fusion (clothianidin and deltamethrin) and Sumishield (clothianidin). Multi-country field trials performed across Africa have confirmed the long-lasting effects of Fludora Fusion and Sumishield on various sprayed surfaces and their efficacy against pyrethroid-resistant malaria vectors, as well as the efficacy of their non-pyrethroid ingredients [72,73,74,75]. However, the recent observations of resistance towards neonicotinoids (e.g., clothianidin and acetamipirid) in Cameroonian *An. gambiae* populations exposed to agricultural clothianidin and acetamiprid [76,77] suggest that it is only an issue of time before neonicotinoid resistance spreads and is established. The recent detection of potential clothianidin resistance in the field populations of *An. funestus* across Africa and the recovery of more susceptibility using piperonylbutoxide synergist assay [78] is in line with our finding that *CYP6P9a* metabolises this insecticide.

Taken together, this evidence, along with the previous studies suggests that these duplicated P450s, *CYP6P9a* and *CYP6P9b*, are the major insecticide resistance-conferring detoxification genes in the major malaria vector, *An. funestus*. Together, they can biotransform insecticides from four different classes in use for public health control of malaria vectors.

## 4. Materials and Methods

### 4.1. Polymorphism Analysis of Full-Length An. funestus CPR cDNA

The genomic samples utilized in this study were from blood-fed, female *An. funestus* caught resting indoors in Malawi, Southern Africa [79] and Uganda, Eastern Africa [80]. The resistance profiles of these mosquitoes have been described in the above publications. To investigate potential genetic variability, *AfCPR* sequences from the above countries were compared to that of the fully susceptible laboratory colony, FANG, which originated from Angola [81].

Total RNA was extracted from five replicates each of 10 female *An. funestus*, which survived deltamethrin exposure, as well as from the female FANG of the same age. The RNA was extracted using the Applied Biosystems Arcturus PicoPure RNA isolation kit (Applied Biosystems, Waltham, MA, USA) according to the manufacturer’s instructions. RNA concentration and integrity were established using Agilent Tape Station (Agilent Technologies, Santa Clara, CA, USA). Complementary DNA (cDNA) was synthesised by reverse transcription, from 1 µg of the extracted RNA, using the SuperScript III (Invitrogen, Waltham, MA, USA) with oligo-dT20 and RNAse H (New England Biolabs, Ipswich, MA, USA). Full-length open reading frames of *AfCPR* were amplified from each cDNA separately using the Phusion Hot Start II High-Fidelity DNA Polymerase (ThermoFisher Scientific, Waltham, MA, USA), with the Agam_AfunCPR primer sets listed in Table S1. In a total volume of 14 µL PCR mix composed of 5X Phusion HF Buffer (containing 1.5 mM MgCl_2_ in final reaction), 85.7 µM dNTP mixes, 0.34 µM each of forward and reverse primers, 0.015 U of Phusion Hot Start II High-Fidelity DNA Polymerase (ThermoFisher Scientific, Waltham, MA, USA). and 10.71 µL of ddH_2_0, 1 µL cDNA was added. Thermocycling conditions were 98 °C for 1 min; followed by 35 cycles each of 98 °C for 20 s (denaturation), 63 °C for 30 s (annealing), extension at 72 °C for 3 min, and a final extension at 72 °C for 10 min; and a hold at 10 °C. The PCR products were gel purified with QIAquick^®^ Gel Extraction Kit (QIAGEN, Hilden, Germany) and ligated into pJET1.2/blunt cloning vector using the CloneJET PCR Cloning Kit (ThermoFisher Scientific, Waltham, MA, USA). These were then cloned into the *E. coli, DH5α* and miniprepped using a QIAprep^®^ Spin Miniprep Kit (QIAGEN, Hilden, Germany). Minipreps were sequenced on both strands using the pJET1.2/blunt forward and reverse sequencing primers.

cDNA polymorphisms were detected through manual examination of sequence traces using BioEdit version 7.7.1 [82] and sequence differences in multiple alignments using CLC Sequence Viewer 6.9, http://www.clcbio.com/ (accessed on 5 October 2023). Different haplotypes were compared by constructing a phylogenetic maximum likelihood tree using MEGAXI [83]. The genetic parameters of polymorphism, including the number of haplotypes (h) and their diversity (Hd), number of polymorphic sites (S), and nucleotide diversity (π), were computed using DnaSP 6.12.03 [84].

### 4.2. Sequence Characterisation of CPR

To identify the functional domains of *AfCPR* [GenBank: EF152578, [85]], the most predominant amino acid sequence (Malawi clone) was compared to those of the closely related species. These include *AgCPR* [GenBank: AY18963375, [8]]; the *An. minimus CPR* [GenBank: EF0957735 [56]], hereafter *AmCPR*; and *An. stephensi CPR* [GenBank: BK008720, [86]], hereafter *AstCPR.* Sequences from the above species were compared to the well-characterised *Rattus norvegicus* (rat) *CPR* [41,42,43] to predict differences in the FMN-binding domain, connecting domain, FAD-binding domain, and NADPH-binding domain. In addition, putative binding domains and catalytic residues were predicted using the InterPro Pfam 36.0 server [87]. Transmembrane-spanning regions were predicted by using the deep learning neural network online tool Deep TransMembrane Hidden Markov Model [DeepTHMM, (DTU/DeepTMHMM—BioLib)].

### 4.3. Comparative Prediction of CPR 3D Folding and Interaction with AfCYP6P9a

To investigate differences in the 3D conformation of *AfCPR* and *AgCPR*, models of the proteins were created. The homology models were built and energy minimised using PyMod 3.0.2 [88], a Schrödinger PyMOL 2.5.8 [89] plugin for MODELLER 10.5 [90]. The models were generated utilizing as a template the crystal structure of rat *CPR* (PDB ID: 1AMO) [41], sharing ~58.15% and ~58.33% similarities, respectively, for *AfCPR* and *AgCPR*, with optimization and refinement levels set as default, objective function, colouring and random seed set to default, and the number of parallel jobs suppressed. A total of 12 models were generated for each sequence, and in addition to internal structural assessments with DOPE and Ramachandran energetic validation, the models were assessed externally using Errat (version 2) to identify the best model from statistical patterns of non-bonded interaction between different atom types [91].

To predict the potential amino acid residues involved in binding to cytochrome P450s, a web-based protein-protein docking server, ClusPro [92] was used to dock the AfCPR and AgCPR models onto the CYP6P9a model. *CYP6P9a* was amplified from Malawi *An. funestus* populations described in the previous study [93] and its amino acid sequence utilized alongside the crystal structure of human *CYP3A4* (36.5% similarity), as a template, to generate its models utilizing PyMod, followed by Errat assessment to select the best 3D structure.

Protein−protein residues non-bonded interactions were analysed using the standalone webtool PDBsum [94], and figures were prepared using PyMOL 2.5.8 [89].

### 4.4. Preparation of Recombinant AfCPR for Heterologous Expression

**Figure 6:**
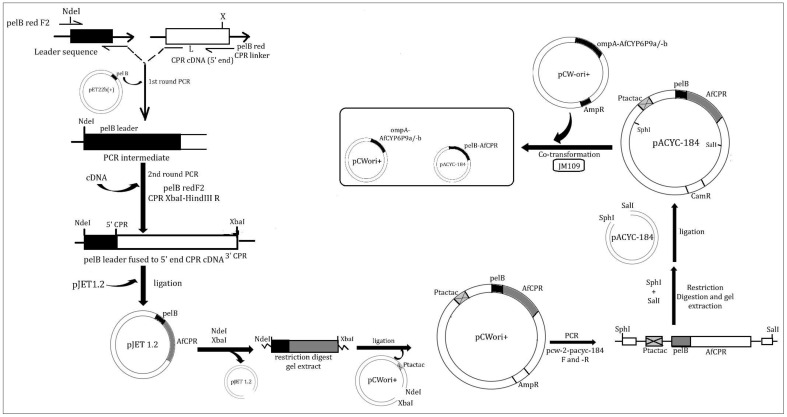
Preparation of recombinant AfCPR with *pelB* leader and *tac-tac* promoter for expression in pACYC-184.

The predominant cDNA of *AfCPR* was prepared for protein expression following the established protocols of Pritchard and colleagues [95,96], with some modifications. Details of the cloning approach are outlined below and presented in Figure 6.

#### 4.4.1. Fusion of AfCPR cDNA to *pelB* Leader

The *AfCPR* sequence was prepared by fusing the bacterial *pelB* leader sequence to the NH_2_-terminus of the *AfCPR* coding sequence, in frame with its initiation codon. In this primary PCR, the forward primer pelB-red-F2, composed of 22 bases of the *pelB* leader and bearing an *Nde*I restriction site (Table S1), was utilised. This targets the 66 nucleotides (22 amino acids) leader peptide from the *pelB* gene encoding pectate lyase B of *Erwinia carotovora* EC [97]. The reverse primer, pelB-red-CPRlinker2, comprises sequences complementary to 20 bases of the *AfCPR* cDNA (5’ end) joined to the last 18 bases (3’ end) of the leader sequence, which allows fusion of the *AfCPR* fragment to the *pelB* leader (Figure 6). The *pelB* leader was copied using pET-22b (+)-Novagen (Merck KGaA, Darmstadt, Germany) as a genomic DNA source. The PCR reaction mix and thermocycling conditions were identical to the protocol outlined in Section 4.1, except the extension and final extension at 72 °C were set to 1 min and 5 min, respectively, and 5X GC buffer was utilised instead of 5X HF buffer.

The linker amplicon was cleaned with a QIAquick^®^ PCR Purification Kit (QIAGEN, Hilden, Germany) and used in a limiting concentration (50 ng) in a secondary PCR, together with *CPR* cDNA, to create the *BamH*I-*Nde*I-*pelB*-*CPR*-*Xba*I-*Hind*III construct, in the presence of pelB-red-F2 primer and a reverse primer (CPR-XbaI-HindIII-R). The PCR mix comprises 5X Phusion HF Buffer, 85.7 µM of dNTP mixes, 0.34 µM each of the above forward and reverse primers, 0.015 U of Phusion Hot Start II High-Fidelity DNA Polymerase (ThermoFisher Scientific, Waltham, MA, USA), 9.21 µL of ddH_2_0, 1 µL of *CPR* cDNA, and 0.5 µL of the linker. Thermocycling conditions were identical to Section 4.1. The PCR products were cleaned with a QIAquick^®^ PCR Purification Kit (QIAGEN, Hilden, Germany) and ligated into the pJET1.2/blunt cloning vector using the CloneJET PCR Cloning Kit (ThermoFisher Scientific, Waltham, MA, USA). These were cloned into *E. coli DH5α*, positive colonies miniprepped with the QIAprep^®^ Spin Miniprep Kit (QIAGEN), and plasmids sequenced on both strands using the pJET1.2 primers.

#### 4.4.2. Cloning of *tac-tac* Promoter into pelB-AfCPR Construct

Because the target expression vector pACYC-184 [98,99] does not harbour an IPTG-inducible promoter for the expression of the *CPR*, this promoter must first be added to the *pelB*-reductase via intermediate subcloning into pCWOri+ [95,96]. However, in contrast to the approach of Pritchard and colleagues (where the *pelB-CPR* constructs cloned into pCWori+ were released from this vector by a *Bcl*I-*Bgl*II double digest and inserted into the *BamH*I site of pACYC-184, abolishing its tetracycline resistance gene), we opted for subcloning of the *pelB-CPR* constructs into the pCWori+ via *Nde*I and *Xba*I, followed by PCR amplification of a fragment flanking the construct plus *tac-tac* promoter [100] of the pCWori+.

Briefly, the plasmids from Section 4.4.1 were digested with FastDigest *Nde*I and *Xba*I (ThermoFisher Scientific, Waltham, MA, USA) restriction enzymes. The digested products were gel-extracted and ligated overnight into *Nde*I- and *Xba*I-linearised pCWori+ vector, creating pB13(*tac-tac*)::*pelB*-*CPR* constructs. These constructs were cloned into *DH5α*, then positive colonies were screened with seqPCWF and seqPCWR primers encompassing the *tac-tac* promoter of pCWori+ (2493 bp fragments) and miniprepped.

#### 4.4.3. Subcloning of *tac-tac*-*pelB*-*AfCPR* Construct into pACYC-184 Expression Vector

To facilitate copying of the above construct for downstream cloning into the expression vector pACYC-184 (Nippon Gene Co. Ltd., Fujifilm, Wako Chemicals, Osaka, Japan), primer sets pcw-2-pacyc-184-F and -R, bearing unique restriction sites (*Sph*I and *Sal*I), were created. The primers copied the pB13(*tac-tac*)::*pelB*-*CPR* fragments from the constructs in Section 4.4.2 for downstream subcloning into the pACYC-184 vector. The PCR conditions are identical to Section 4.1, except for the final extension which was set to 15 min at 72 °C, to allow enough time for the amplification of this 2786 bp fragment. The PCR amplicon was purified as described above, digested successfully with FastDigest *Sph*I and *Sal*I, gel extracted, and ligated overnight with pACYC-184 vector linearized with the same restriction enzymes. Ligation products were transformed into *DH5α*. Colonies were screened successfully with two sets of primers. In addition to PCR screening with the above pcw-2-pacyc-184 primers, which confirm a fragment of ~2800 bp (insertion in pCWori+), additional PCR was carried out using seqCPRF2 (an internal primer designed within the *CPR* gene, to amplify the 1133 bp fragment) and seqpACYC-184R (a reverse complement internal primer designed within the pACYC-184 vector, 73 bp downstream the *Sal*I restriction site). Positive colonies screened with these second primers produced a gel band of 1206 bp, confirming the presence of *CPR* within pACYC-184. These plasmids were then miniprepped.

### 4.5. Heterologous Co-Expression of Recombinant AfCYP6P9a/-b with AfCPR and AgCPR

The above pACYC-AfCPR plasmid construct was co-transformed together with either *An. funestus CYP6P9a* (*CYP6P9a*) or *An. funestus CYP6P9b* (*CYP6P9b*) into *E. coli JM109*. These two pyrethroid-metabolizing specialist P450s were amplified from cDNA from mosquitoes described above [93], and prepared as detailed in our previous studies [19,20]. Membrane expression and preparation follow the procedure of Pritchard [95], with the modifications we have published in several studies [19,20]. To compare the potential impact of the endogenous *AfCPR* on the metabolism of insecticides, the above P450s were also co-expressed together with the *AgCPR*, as we have done previously [19,20,37]. The recombinant CYP450s expressed optimally at 21 °C and 150 rpm, 36–40 h after induction with 0.5 mM δ-ALA and 1 mM IPTG to the final concentrations. The membrane contents of the P450s were determined spectrally [101].

To account for potential differences in metabolic activities which can be attributed to differences in the concentrations of P450s and CPRs in the membranes, protein concentrations were established using Bradford assays [102]. Following the Bradford assays, the protein contents of the membranes were normalized by dilution in 1X TSE buffer, adjusting the concentrations of all four membranes to 20.8 mg/mL, followed by determination of spectral activity again.

### 4.6. Comparative Determination of CPR Activities Using Model Substrate Cytochrome c

The activities of the recombinant CPR proteins in the purified membranes were assayed by monitoring increased absorbance at 550 nm in the presence of electron acceptor *cytochrome c*. Briefly, for each membrane, a 300 µL reaction mixture containing 0.1 mM horse heart *cytochrome c* (dissolved in potassium phosphate buffer, pH 7.7) and 2 µL of purified recombinant membrane were added into a 96-well ELISA plate. The reactions were initiated by adding 0.1 mM NADPH (dissolved in the same buffer). The time-dependent absorption increase in samples was monitored on a BioTek Epoch 2 microplate reader (Agilent, Santa Clara, CA, USA). The recombinant CPR activities were calculated from the *cytochrome c* reduced using the formula of Guengerich and colleagues [103]. Reactions were performed in triplicates both for +NADPH and –NADPH (controls). Kinetic parameters *K_m_* and *V_max_* were calculated from the Michaelis–Menten plot using the least squares, non-linear regression in the GraphPad Prism 5.0 (GraphPad Inc., La Jolla, CA, USA). Kinetic analyses were carried out using the same protocol as above but with eight serially diluted *cytochrome c* concentrations (6.25–300 µM).

### 4.7. Comparative Determination of Insecticides Metabolizing Activities

To investigate the potential impact of indigenous *AfCPR* on *CYP6P9a* and *CYP6P9b* metabolism of public health insecticides, in vitro assays were carried out using the recombinant proteins co-expressing the P450s and their *AfCPR*, side-by-side with the same P450s co-expressed with *AgCPR*. Substrate depletion assays were conducted with type I pyrethroid (permethrin), type II pyrethroids (deltamethrin and α-cypermethrin), an organochloride (DDT), a carbamate (bendiocarb), neonicotinoids (clothianidin, imidacloprid and acetamipirid), and a pyrrole (chlorfenapyr). The protocols for incubation and high-performance liquid chromatography (HPLC) analyses for the above insecticides followed procedures previously published [21,23,63,104], with some modifications.

The assay mix comprised 0.45 µM membrane expressing recombinant proteins, 1.8 µM reconstituted cytochrome b_5_ protein (4:1 ratio), and 20 µM insecticide, diluted in 100 µL water. Membranes containing the recombinant proteins, cytochrome b_5,_ and insecticides were added to the side of the tubes. The reactions were started by adding 100 µL of 1 mM final concentration of NADPH regeneration buffer. The NADPH regeneration buffer contained 1 mM glucose-6-phosphate, 0.1 mM NADP^+^, 0.25 mM MgCl_2,_ and 1 unit/mL glucose-6-phosphate dehydrogenase prepared in 50 mM phosphate buffer, pH 7.4. Negative reactions contained the above regeneration buffer mix but without the NADP^+^ (equal volume of buffer added in place of the amount of the buffer containing NADP^+^). Reactions were conducted in triplicate for positive and negative incubations for each insecticide. Following incubation for 2 h at 30 °C and 1200 rpm, reactions were quenched with 200 µL of ice-cold acetonitrile and samples were incubated for an additional 10 min to dissolve the insecticides, before centrifugation at 20,000× *g* for 20 min. The supernatants (100 µL each) were loaded into the HPLC vials and 50 µL injected into the isocratic mobile phase, with a flow rate of 1 mL/min, and peaks separated with 250 mm C18 column (Acclaim 120^TM^, Dionex) on Agilent 1260 Infinity.

For pyrethroids, peaks were separated at 23 °C and 226 nm, with a 70:30 acetonitrile:water mobile phase, and retention times set to 30 min. For DDT, 1 mM of solubilizing factor sodium cholate was added to the incubation mix [14,104] and peaks were separated at 23 °C and 232 nm, with a retention time of 20 min. For bendiocarb, separation was achieved with a 65:35 acetonitrile:water mobile phase, at 205 nm, with retention time set to 20 min and column temperature set to 40 °C [21,23]. For neonicotinoids, separations were achieved at a wavelength of 260 nm for clothianidin, 272 nm for imidacloprid, and 220 nm for acetamiprid, on 15:85 acetonitrile:water (containing 0.1% H_3_P0_4_), with retention time set to 20 min and column temperature set to 40 °C. Chlorfenapyr was detected at 23 °C and a wavelength of 260 nm, using 70:30 methanol:water (containing 0.1% H_3_P0_4_), with retention time set to 20 min and a column temperature of 35 °C. Enzyme activity was calculated as percentage depletion (the difference in the amount of insecticide(s) remaining in the +NADPH tubes, compared with the –NADPH) and a Student’s t-test was used to test for statistical signification.

Steady-state kinetic parameters were determined with DDT by measuring the rate of reaction under linear conditions for 1 h while varying the substrate concentrations (1.25–20 µM) in the presence of 45 pmol each of the recombinant AfCYP6P9a-AfCPR. For each DDT concentration, the reactions were performed in triplicate both for +NADPH and –NADPH. *K_m_* and *V_max_* were established from the plot of substrate concentrations against the initial velocities and fitting of the data to the Michaelis–Menten module using least squares non-linear regression in GraphPad Prism 6.03 Software (GraphPad Inc., La Jolla, CA, USA).

## 5. Conclusions

Resistance risk assessment is important in public health insecticide evaluation schemes, and knowledge of resistance dynamics of novel insecticide-based control tools is essential for anticipating and strategizing to manage resistance, thereby extending the lifespan of insecticides.

An important toolbox to characterise resistance and its molecular basis is *in vitro* functional genomics. This approach has been applied to characterise resistance genes, particularly the CYP450s from the Anopheles malaria vectors.

In this study, we have shown that the endogenous *AfCPR* not only increased pyrethroid metabolism by the two key resistance-associated genes, *CYP6P9a* and -*b* but also enabled the former P450 to metabolise non-pyrethroid insecticides. This includes DDT and bendiocarb (important ingredients in use for indoor residual spraying), as well as chlorfenapyr and clothianidin, which are novel insecticides in use for impregnation of next-generation long-lasting insecticidal bed nets and indoor residual spraying, respectively. Future studies which seek to validate the role of P450s in *An. funestus*, other Anopheles mosquitoes, and other insects should endeavour to characterise the P450s in tandem with their endogenous P450 reductases.

## Figures and Tables

**Table 1:** Summary statistics for polymorphisms of AfCPR.

Population	n	S	Syn	Non syn	h	H_d_	π (k)	D (Tajima)	D * (Fu and Li)
MALAWI	5	0	0	0	1	0.00	0.00	0.00	0.00
UGANDA	5	9	5	4	2	0.60	0.0027	1.78	1.78 *
FANG	5	0	0	0	1	0.00	0.00	0.00	0.00
All	15	252	222	33	4	0.77	0.058	2.30 *	1.73 **

n = number of sequences (n); S, number of polymorphic sites; h, haplotype; H_d_, haplotype diversity; Syn, Synonymous mutations; Non syn, Non-synonymous mutations; π, nucleotide diversity (k = mean number of nucleotide differences); Tajima’s D and Fu and Li’s D statistics, s, significant: * *p* < 0.05; ** *p* < 0.02.

## Data Availability

The CPR cDNA sequences reported in this paper were deposited in the GenBank, with accession numbers PP503425-PP503439.

## Supplementary Materials

The following supporting information can be downloaded at: https://www.mdpi.com/article/10.3390/ijms25158092/s1.
