# Supplementary material for: Functional Validation of Endogenous Redox Partner Cytochrome P450 Reductase Reveals the Key P450s CYP6P9a/-b as Broad Substrate Metabolizers Conferring Cross-Resistance to Different Insecticide Classes in Anopheles funestus
Source: Int J Mol Sci. 2024 Jul 25;25(15):8092. doi: 10.3390/ijms25158092 (PMC11311542; doi:10.3390/ijms25158092)
Supplement: Supplementary file 1 [file ijms-25-08092-s001.zip › Table S1.pdf]

Table S1: List of primers used for amplification and functional characterisation of *CPR*.

Gene	Forward Primer	Reverse Primer	Amplicon size (bp)
Agam_AfunCPR	ATGGACGCCCAGACAGAAAC	TTAGCTCCACACGTCCGCC	2,040
pelB-red-F2	GGATCCCATATGAAATACCTGCTGCCGACCG		
pelB-red-CPRlinker2		GTTTCTGTCTGGGCGTCCAT GGCCATCGCCGGCTGGGC	86
CPR-XbaI-HindIII		TTCGAAAGATCTTTAGCTCCACACGTCCGCC	2,106
seqpCWF	ATCCCCCTGTTGACAATTAATCATC		
seqpCWR		ACCTATAAAAATAGGCGTATCACGA	2,493
pcw-2-pacyc-184F-BspHI-SphI	TCATGAGCATGCGATAAGAGACACCGGCATACTCTGC		
pcw-2-pacyc-184R-EagI-SalI		GTCGACCGGCCGGAGGCCCTTTCGTCTTCAAGCA	2,786
seqCPRF2	GCATGCACGTCGAGTTCGAT		
seqpACYC-184R		GGCGACGATAGTCATGCCC	1,206

Pink is *BamH*I, Red is *Nde*I, and light green is the *pelB* leader nucleotides in frame with the start codon from *Nde*I. *pelB* 3' end (reverse complement) is in turquoise and underlined is *An. funestus* or *An. gambiae* NH_2-terminus 20 nucleotides (reverse complement). Yellow is *Xba*I, Grey-50 is *Hind*III, Blue is *Sph*I, Dark green is *Sal*I.
