# Supplementary figures and images for: Functional Validation of Endogenous Redox Partner Cytochrome P450 Reductase Reveals the Key P450s CYP6P9a/-b as Broad Substrate Metabolizers Conferring Cross-Resistance to Different Insecticide Classes in Anopheles funestus
Source: Int J Mol Sci. 2024 Jul 25;25(15):8092. doi: 10.3390/ijms25158092 (PMC11311542; doi:10.3390/ijms25158092)

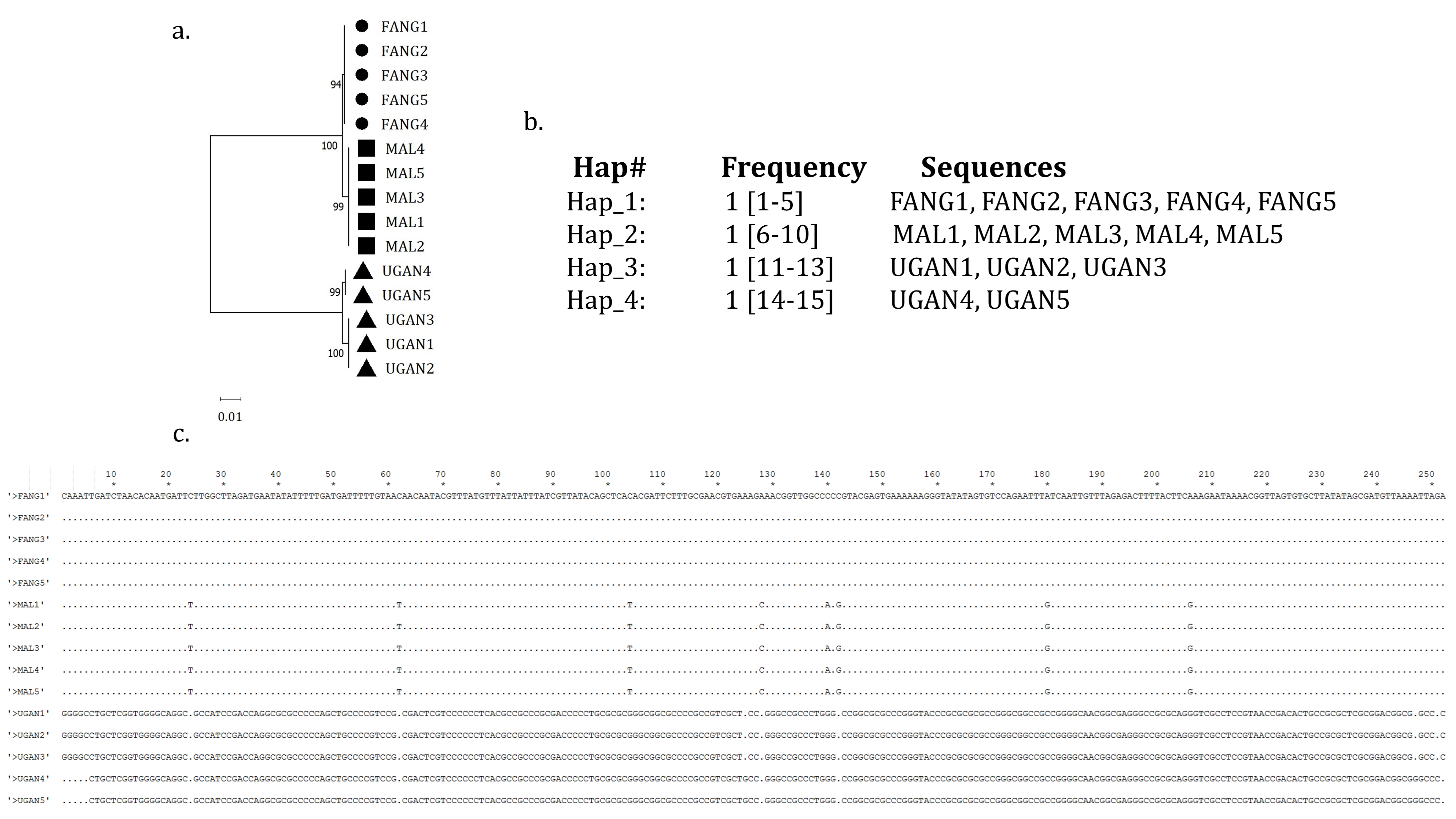

Supplement: Supplementary file 1 [file ijms-25-08092-s001.zip › Figure S1-Ibrahim et al 2024 ijms.tif]

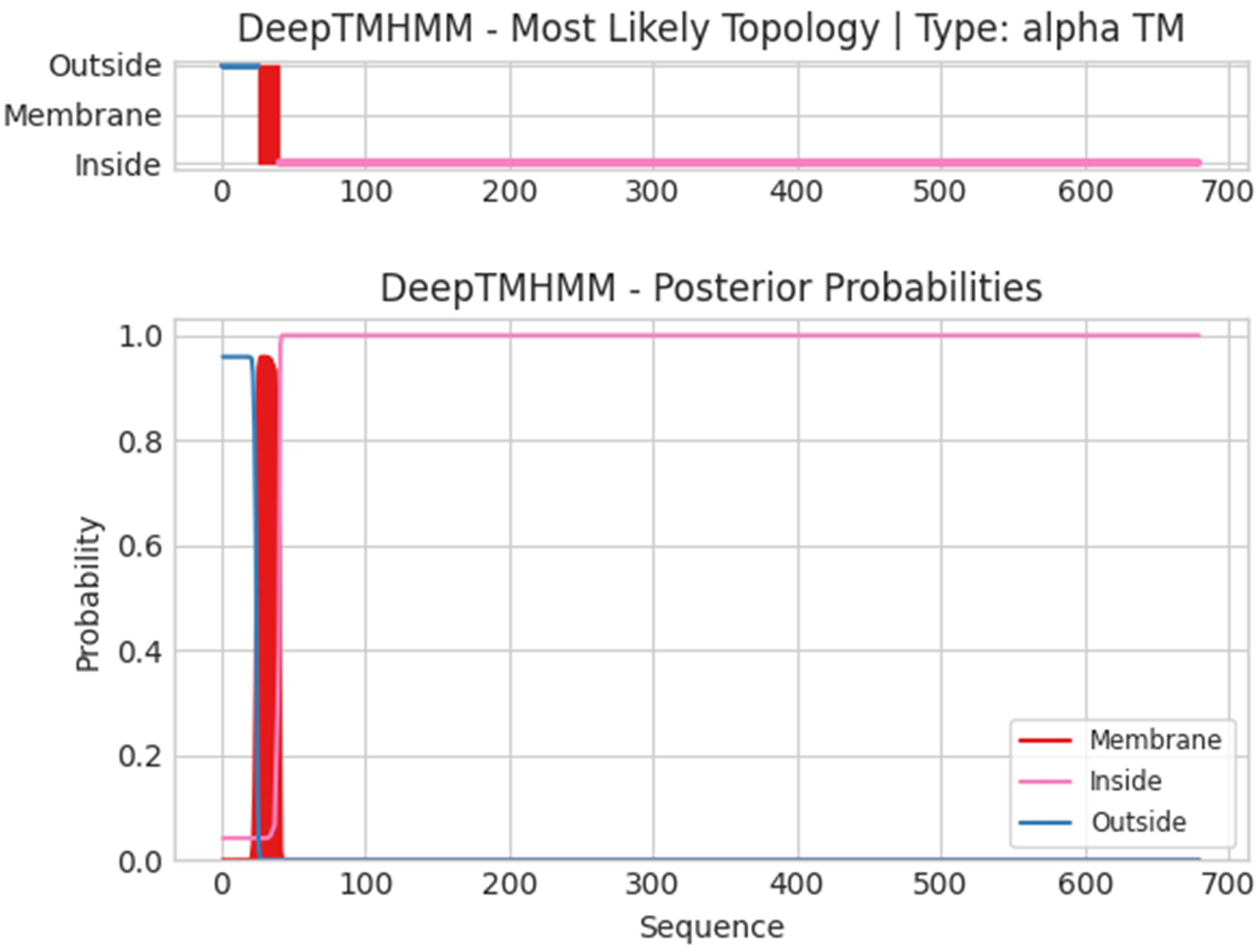

Supplement: Supplementary file 1 [file ijms-25-08092-s001.zip › Figure S2-Ibrahim et al 2024 ijms.tif]

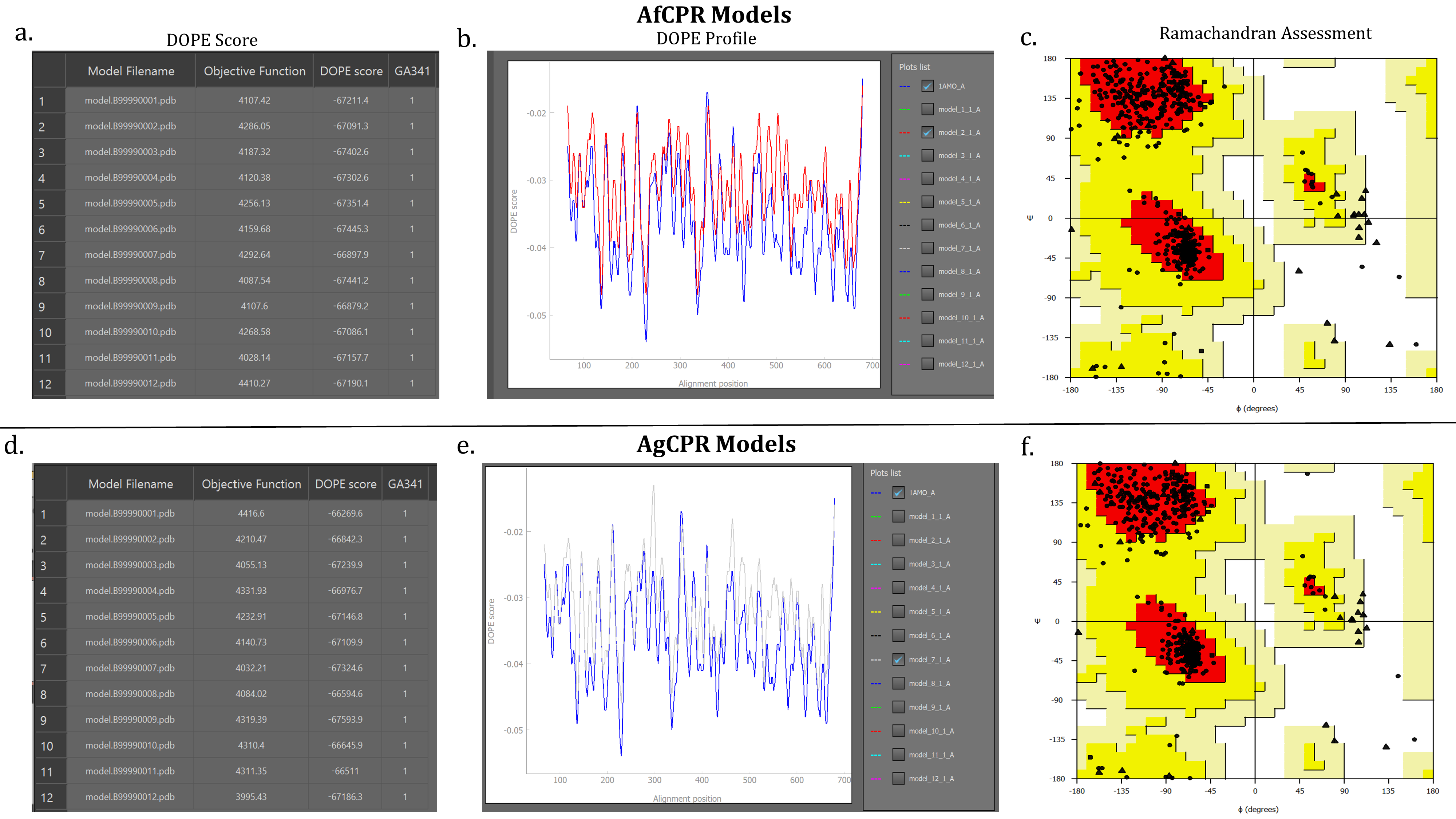

Supplement: Supplementary file 1 [file ijms-25-08092-s001.zip › Figure S3-Ibrahim et al 2024 ijms.tif]

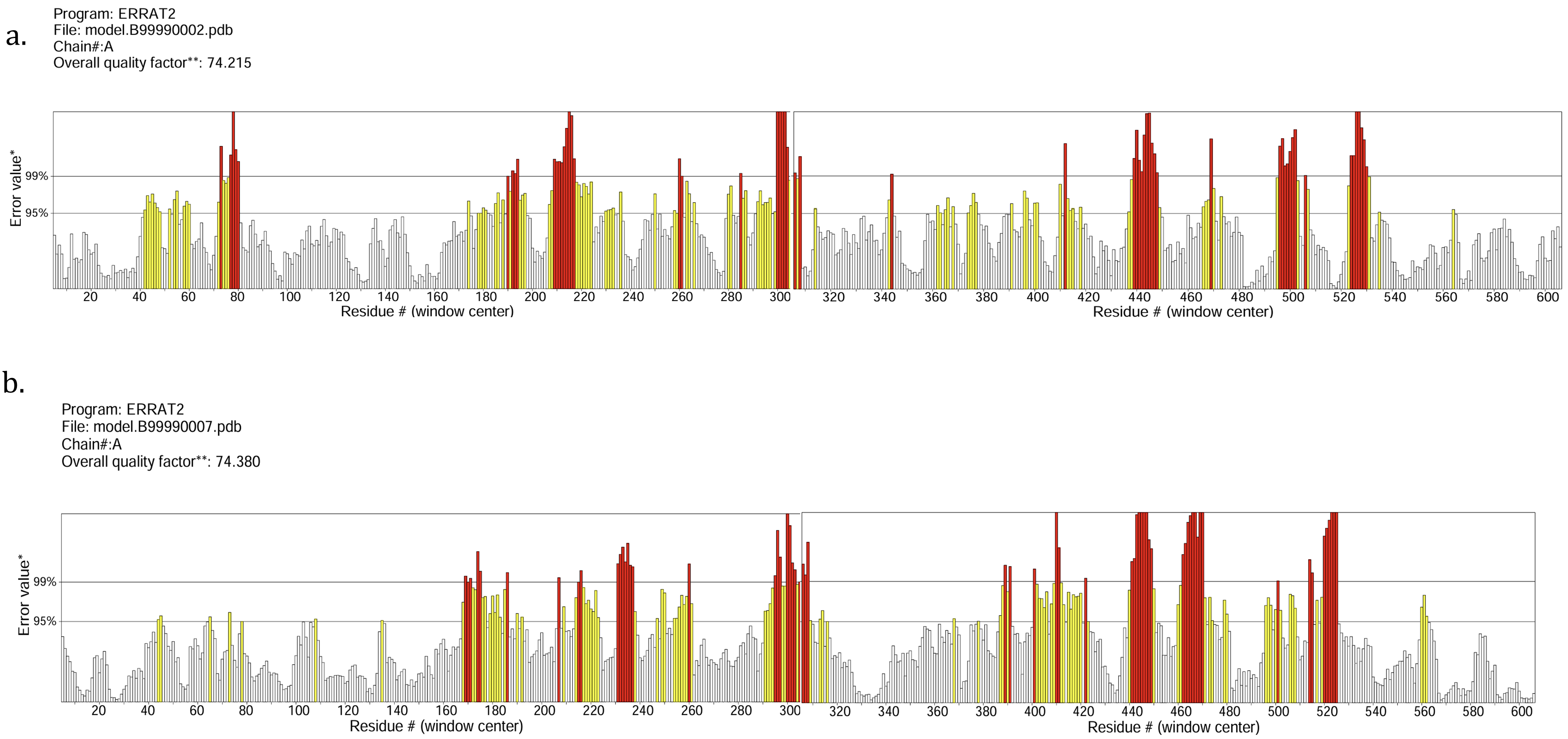

Supplement: Supplementary file 1 [file ijms-25-08092-s001.zip › Figure S4-Ibrahim et al 2024 ijms.tif]

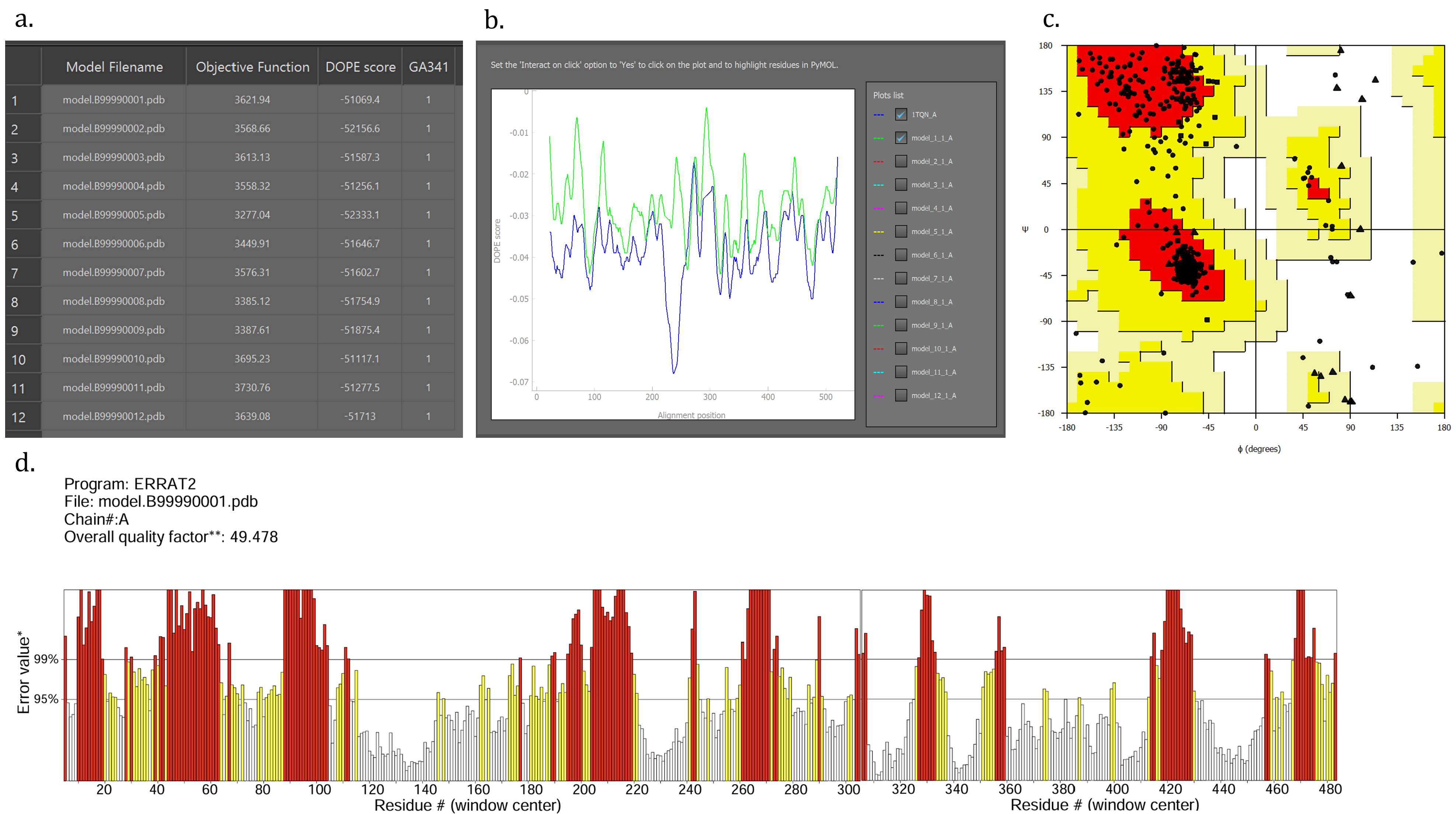

Supplement: Supplementary file 1 [file ijms-25-08092-s001.zip › Figure S5-Ibrahim et al 2024 ijms.tif]

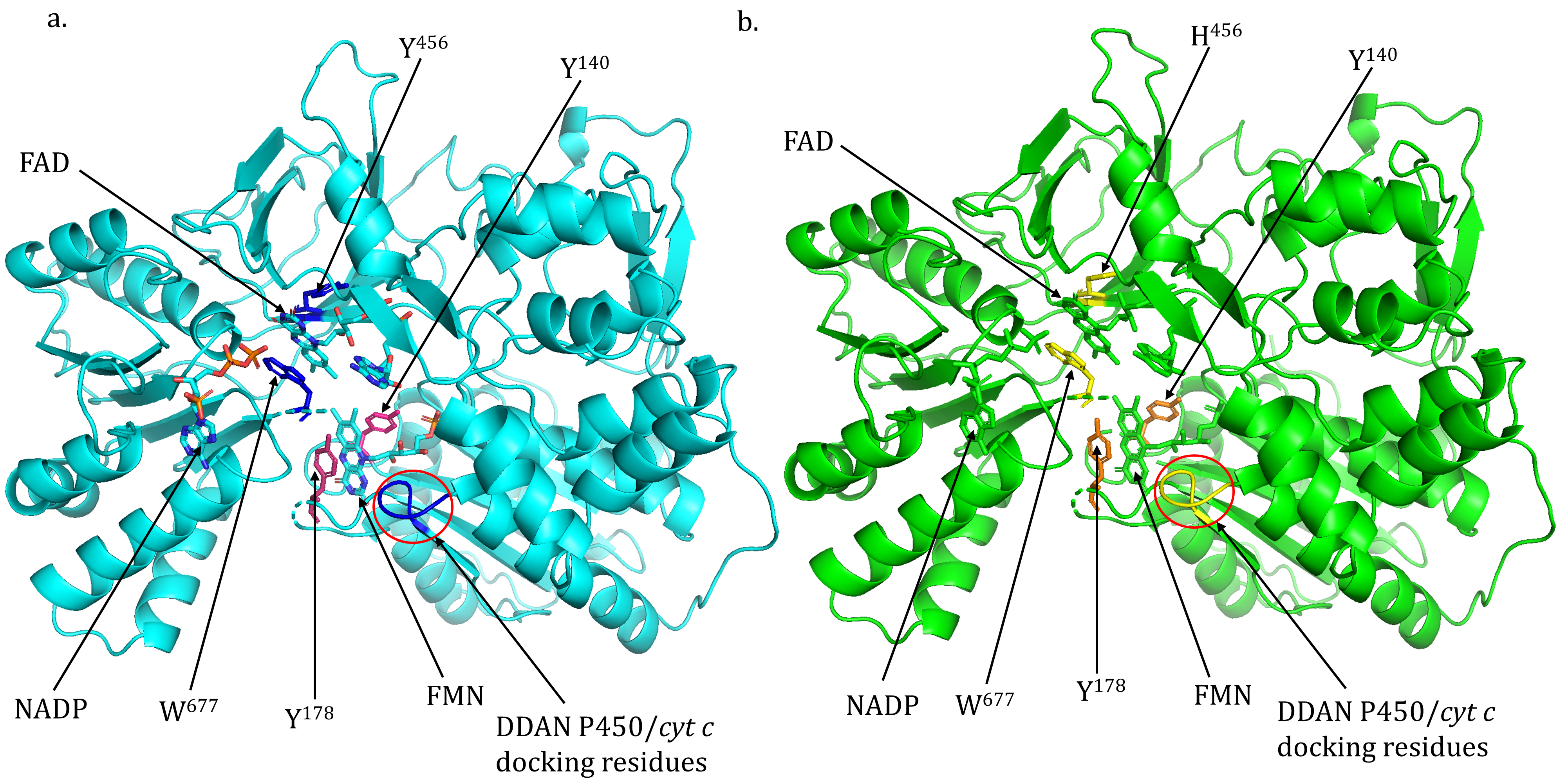

Supplement: Supplementary file 1 [file ijms-25-08092-s001.zip › Figure S6-Ibrahim et al 2024 ijms.tif]
